# Clinicopathologic features and responses to radiotherapy of myeloid sarcoma

**DOI:** 10.1186/1748-717X-8-245

**Published:** 2013-10-22

**Authors:** Wan-Yu Chen, Chun-Wei Wang, Chin-Hao Chang, Heng-Hsiu Liu, Keng-Hsueh Lan, Jih-Luh Tang, Hwei-Fang Tien, Sung-Hsin Kuo, Ann-Lii Cheng

**Affiliations:** 1 Division of Radiation Oncology, Department of Oncology, National Taiwan University Hospital and National Taiwan University College of Medicine, Taipei, Taiwan; 2 Department of Medical Research, National Taiwan University Hospital and National Taiwan University College of Medicine, Taipei, Taiwan; 3 Department of Internal Medicine, National Taiwan University Hospital and National Taiwan University College of Medicine, Taipei, Taiwan; 4 National Translational Medicine and Clinical Trial Resource Center, Taipei, Taiwan; 5 Cancer Research Center, National Taiwan University College of Medicine, Taipei, Taiwan; 6 Graduate Institute of Oncology, National Taiwan University College of Medicine, Taipei, Taiwan; 7 Graduate Institute of Clinical Medicine, National Taiwan University College of Medicine, Taipei, Taiwan

**Keywords:** Chloroma, Granulocytic sarcoma, Myeloid sarcoma, Extramedullary leukemia, Radiotherapy

## Abstract

**Background:**

To evaluate clinicopathological features, radiotherapeutic parameters, and their associations with responses to radiotherapy (RT) in patients with myeloid sarcoma (MS).

**Methods:**

We reviewed 20 patients receiving RT for MS lesions (in 43 RT courses) and analyzed the patients’ clinicopathologic features and radiotherapeutic parameters, and their associations with complete responses (CR) to RT using Fisher’s exact test and univariate logistic regression analysis. Generalized Estimating Equation was used to analyze all 43 irradiated lesions and account for the correlations in RT responses among lesions from the same patient.

**Results:**

We found that the underlying hematological diseases of the evaluated patients were acute myeloid leukemia (AML) in 14 patients (70%), chronic myeloid leukemia in 4 patients (20%), myelodysplastic syndrome with AML transformation in one patient (5%), and de novo MS in one patient (5%). Most patients (55%) received RT for MS at the time of relapse following bone marrow transplantation (BMT). The most common cytogenetic abnormality was t(8;21)(q22;q22). The median RT dose of 20 Gy (range 6–35 Gy), administered in 1.5-3.5 Gy fractions, provided a 63% CR rate. RT dose, sex, cytogenetics, and bone marrow status at the time of RT had no significant effect on CR. Younger age (<50 y, *P* = 0.06), BMT prior to RT (*P* = 0.05), and underlying AML (*P* = 0.05) were marginally associated with higher CR to RT.

**Conclusions:**

Our results indicate that a modest RT dose (20-30 Gy) achieves good local control of MS. Age, previous BMT, and underlying hematologic disease can affect RT response.

## Introduction

Myeloid sarcoma (MS), also known as granulocytic sarcoma or chloroma, is a rare clinical condition characterized by aggregation of immature myeloid cells presenting as an extramedullary mass [1]. The most commonly involved sites are the small intestine, bone, skin, and lymph nodes [2]. MS can develop de novo or emerge during the clinical course of acute myeloid leukemia (AML), myeloproliferative disorder (MPD), or myelodysplastic syndrome (MDS). In AML patients, MS might represent the first manifestation of AML, predating it by months or years. It can also be the initial presentation of relapse in remission, with or without underlying bone marrow (BM) relapse, with reported incidence of 3-9% [3,4].

MS can present at various sites of the body resulting in prominent symptoms and signs. Diagnosis of MS requires a high degree of vigilance by physicians. Its optimal treatment remains controversial. Chemotherapy or hematopoietic cell transplantation is often considered the frontline treatment for MS. Radiotherapy (RT) could be reserved for palliation of symptomatic or rapidly progressive lesions [5,6] or as part of a combined modality treatment in conjunction with chemotherapy or hematopoietic cell transplantation. RT is also considered a consolidative treatment for isolated MS without BM involvement, or during BM remission after systemic therapy [7]. However, data on outcome from RT in MS is limited and most previous studies are case series reports.

In this study, we investigated the possible underlying predictive factors, including clinicopathologic features and radiotherapeutic parameters, for response to RT in MS patients who received frontline or palliative RT. We evaluated 20 patients and their irradiated lesions, and reviewed responses to RT and RT doses reported in previous studies on MS patients who received frontline or palliative RT.

### Patients and methods

#### Patients and clinical characteristics

Twenty consecutive patients with MS who received RT at our institute from 2000 to 2010 were retrospectively reviewed. This restrospective study was approved by institutional review board of National Taiwan University Hospital. Pathologic evaluation of incision biopsy or needle aspiration of these 20 patients demonstrated the histomorphologic features of MS characterized by homogenous malignant infiltrations of immature large cells with ovale and slightly nuclei. Further immunohistochemical studies, including lysozyme and myeloperoxidase (MPO), CD68, and chloroacetate, were used to detect their myeloid origins and exclude lymphoreticular tumors and mesenchymal tumors. The histopathological characteristics and immunohistochemical results of all tumor specimens were independently reviewed by experienced hematopathologists. Cytogenetic studies were also performed. The patients had 43 irradiated lesions. Clinicopathologic features including age, sex, underlying hematologic diseases, MS site for RT, timing of MS emergence, cytogenetics, and radiotherapeutic parameters (including RT dose and fractionation), and responses of the treated lesions to RT were assessed. During the same period, 18 MS patients without RT served as control group.

#### Radiotherapy

RT was provided using ^60^Cobalt, 6–10 MV linear accelerators or 9–12 MeV electrons for superficial lesions. The RT field was the gross tumor and a 2–3 cm margin. Radiation doses ranged from 6 Gy to 35 Gy (median, 20 Gy) at a dose per fraction of 1.5-3.5 Gy (median, 2 Gy). Twenty-eight lesions (65.2%) received RT in doses ≥20 Gy. Because various fraction sizes were used, conversion of the total radiation dose to biologically equivalent doses (BED) delivered at 2 Gy per fraction (BED2) were calculated using the linear-quadratic model [6]:

$$\text{BED}2\left(\text{Gy}\right)=\frac{\mathrm{nd}\left(1+\frac{d}{\alpha /\beta }\right)}{\left(1+\frac{2}{\alpha /\beta }\right)}$$

where n is the number of fractions and d is the dose (Gy) per fraction. An α/β ratio of 10 is used for leukemia [7], resulting in a BED2 ranging from 6.5 Gy to 35.9 Gy (median, 20 Gy).

Lesions’ initial responses to RT were assessed by physical examination, computed tomography (CT), or magnetic resonance imaging (MRI) within 4 weeks of completion of RT. A complete response (CR) was defined as no evidence of disease and a partial response (PR) as >50% regression of all measurable tumor mass. Lesions with <50% regression, stable disease (SD), or progression were classified as displaying no response.

#### Statistical analysis

Seven of the 20 patients received multiple courses of RT to distinct lesions, resulting in 43 irradiated lesions. Among 7 patients with multiple courses of RT, 2 patients received coinstantaneous irradiation to multiple sites and 5 patients received multiple sequential courses of RT. If a patient received multiple sequential courses of RT, data from the first RT course were used for analysis. Data from one RT site with coinstantaneous irradiations were randomly selected when patients concurrently received RT to multiple sites. Treatment responsesto RT doses (BED), underlying hematologic diseases, age, sex, cytogenetics, bone marrow transplant (BMT) prior to RT, and BM status at the time of RT in MS were correlated. Fisher’s exact test was used to evaluate the associations between category variables. Crude odds ratios (OR), 95% confidence intervals (CI), and *P*-values were calculated using univariate logistic regression analysis. Post-RT overall survival (OS) was calculated using the Kaplan-Meier method.

The Generalized Estimating Equation(GEE) was introduced by Zeger and Liang [8] to cope with clustering data that would otherwise be analyzed using a generalized linear model. GEE has become an essential tool for the analysis of correlated data. In this study, GEE was used to account for correlations in responses to RT among lesions from the same patient. The unit of analysis was the irradiated lesion. All statistical analysis was performed using SAS software (9.2, SAS Institute Cary, NC).

## Results

### Clinicopathologic features and survival of MS patients with RT

We included 20 patients (median age, 45 years old (y), range 4–83 y; 10 men and 10 women) with 43 irradiated lesions in our analyses. The median follow-up time was 4.3 months (range, 0.5-56.7 months). Table 1 displays the clinicopathologic features of the patients and their treated lesions. The underlying hematologic diseases included AML in 14 (70%) patients, chronic myeloid leukemia (CML) in 4 (20%) patients, MDS with AML transformation in one (5%) patient, and de novo MS in one (5%) patient. Of the 14 AML patients, 6 (42.9%) were French-American-British (FAB) M2, 5 (35.7%) were FAB M1, 2 (14.3%) were FAB M4, and one (7.1%) was FAB M5. The irradiated lesions in the 20 patients included 23 (53.5%) skin, 4 (9.3%) breast, 4 (9.3%) brain parenchyma, 3 (7%) spinal cord, and 3 (7%) bone lesions, plus one (2.3%) anal, one (2.3%) cervical, one (2.3%) pituitary, one (2.3%) paranasal sinus, one (2.3%) buccal mucosal, and one (2.3%) nipple lesion.

**Table 1:** Patient characteristics for MS patients with RT

**Patient no.**	**Age**	**Gender**	**Underlying disease**	**GS site for radiotherapy**	**Timing of GS emergence**	**Cytogenetics**
1	62	F	AML-M2	Skin	Relapse after chemotherapy, BM also (+)	Normal
2	50	M	CML	Anus	CML in blast crisis	47 XXY, inv(11)
3	83	M	AML-M4	Skin	Found at initial presentation with BM (+)	t(2;14), NPM(-), FLT3(-)
4	55	M	AML-M1	Spinal cord	Relapse after chemotherapy, BM also (+)	t(9;22) Philadelphia chromosome (+)
5	58	M	AML-M1	Skin (7 lesions)	Relapse after BMT, BM also (+)	Normal
6	6	F	CML	Bone (right femur)	CML in blast crisis	t(9;22) Philadelphia chromosome (+)
7	71	F	MDS- RAEB 2	Brain	-	Normal
8	41	F	AML-M2	Cervix	Relapse after BMT, BM (-)	t(8;21)
				Right breast	Relapse after BMT, BM (-)	
9	42	M	*de novo* sarcoma	Right nipple	Found at presentation, BM (-)	Normal
				Brain	Relapse after chemotherapy, BM also (+)	
				Paranasosinus	Relapse after chemotherapy, BM also (+)	
10	56	M	CML	Spinal cord	CML in blast crisis	Normal
11	22	M	AML-M4	Skin (11 lesions)	Relapse after BMT, BM (-)	del(1)(p32p36), t(10;11)(p13;q13)
12	44	F	CML	Right breast	CML in blast crisis receiving BMT. relapse after BMT, BM (-)	Normal
13	75	F	AML-M2	Skin	Found at initial presentation with BM (+)	del(15)(q22q35)
14	29	F	AML-M2	Pituitary gland	Relapse after BMT, BM (-)	Normal
15	32	F	AML-M1	Right breast	Relapse after BMT, BM (-)	Normal
				Left breast	Relapse after BMT, BM (-)	
16	47	M	AML-M1	Brain	Relapse after chemotherapy, BM also (+)	Normal
				Spinal cord	Relapse after chemotherapy, BM also (+)	
17	47	F	AML-M2	Skin	Relapse after chemotherapy, BM also (+)	t(8;21)
18	40	M	AML-M5	Skin	Emerging during chemotherapy and persistent after BMT, BM (+)	-3p,t(5;17)(q15;q22), t(11;19)(q23;p13),-17,19p; der(19)t(17;19)(q11;p11), t(11;12)(p15;q13), NPM(-), FLT3(-)
19	42	F	AML-M1	Right inguinal area	Found at initial presentation with BM (+)	Normal
20	4	M	AML-M2	Bone (left tibia)	Relapse after BMT, BM also (+)	t(8;21), AML1-ETO fusion transcription (+)
				Left buccal mucosa	Relapse after BMT, BM also (+)	
				Bone (right foot)	Relapse after BMT, BM also (+)	

*Abbreviations:* AML = acute myeloid leukemia; BM = bone marrow; BMT = bone marrow transplant; CML = chronic myeloid leukemia; FLT3 = FMS-like tyrosine kinase 3; MDS = myelodysplastic syndrome; NPM = nucleophosmin; RAEB = refractory anemia with excess blasts.

In our study, most irradiated MS lesions (17 lesions, 39.5%) were from patients with extramedullary relapse following BMT, and 10 (23.3%) lesions were from patients with BM relapse accompanied by extramedullary relapse. Seven (16.3%) lesions emerged following chemotherapy for underlying BM disease. Three (7%) lesions presented at diagnosis of hematological malignancy with BM involvement, 3 (7%) lesions presented during CML blast crisis, one (2.3%) lesion emerged during induction/consolidation chemotherapy and was persistent after BMT with positive BM disease, and one (2.3%) lesion presented as de novo sarcoma as the sole site without underlying BM disease. Of the 20 patients, 10 (50%) had normal cytogenetics and 10 (50%) displayed abnormal chromosomes, among which t(8;21)(q22;q22) was the most common abnormality (in 3 (30%) of 10 patients). Other observed abnormal cytogenetics included t(2;14), t(9;22), and del(15)(q22q35). Three patients displayed other chromosome complex changes: del(1)(p32p36), t(10;11)(p13;q13) in one patient, -3p, t(5:17)(q15;q22), t(11:19)(q23;p13), -17, +19p; der(19)t(17;19)(q11;p11), t(11;12)(p15;q13) in one patient, and 47 XXY with inv(11) in one patient.

**Figure 1:**
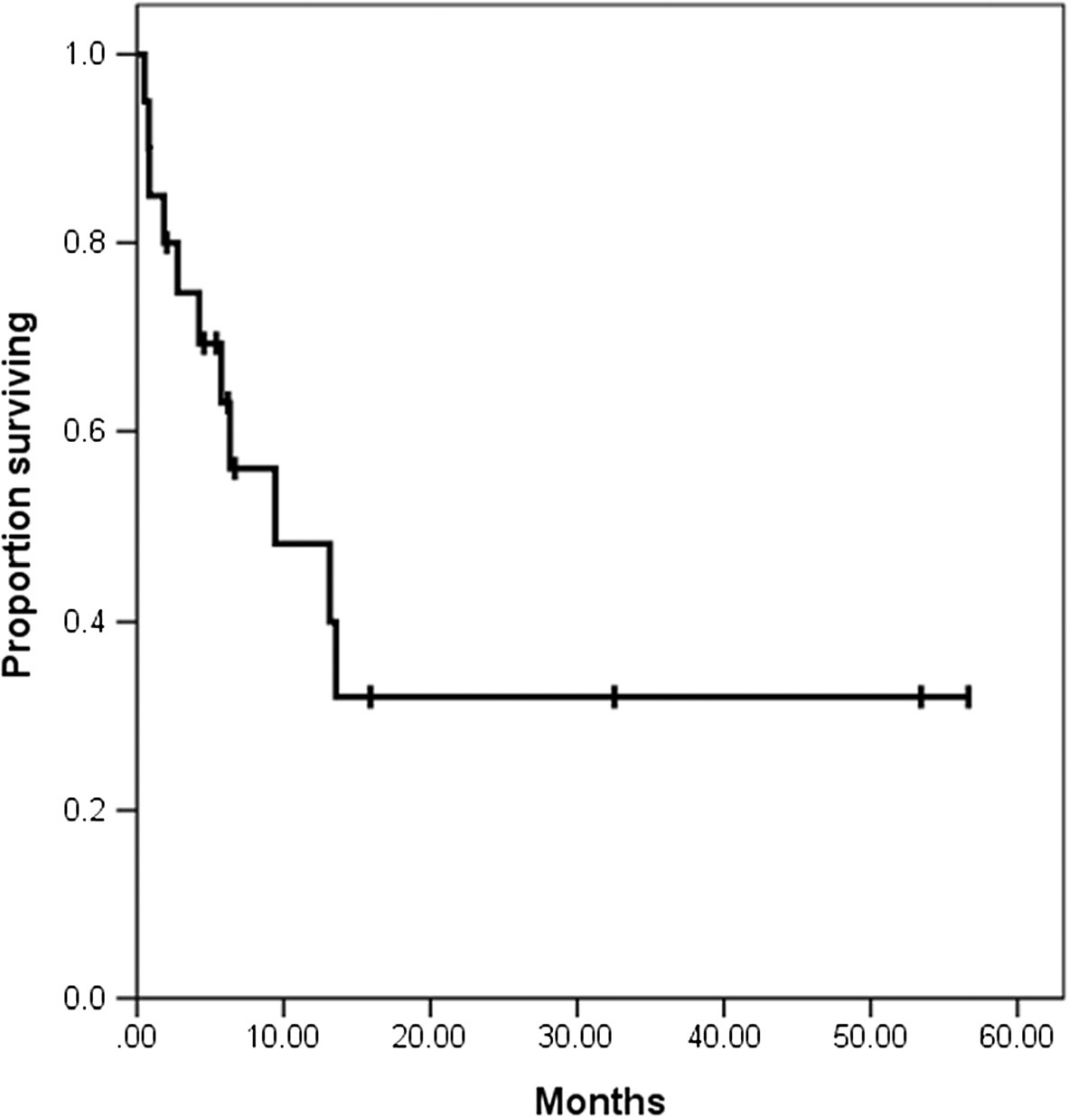
Kaplan-Meier overall survival curve for patients who received RT (radiotherapy) for MS (post RT survival rate).

Of the 20 patients, 9 survived and 11 died. The median survival time following RT was 6 months (range, 0.5-57 month) in all patients and 6.7 months (range, 2–57 month) in the surviving patients. The post-RT 1-year OS was 24% (Figure 1).

### Correlations between RT responses and clinical characteristics

When evaluating the RT responses of the 20 patients with MS, we identified 13 patients (65%) with CR, 5 (25%) with PR, and 2 (10%) with SD. Totally, 19 (95%) of 20 patients achieved symptoms relief of MS after RT, except one patient with SD had persistent symptoms. Among patients with symptoms relief, 6 patients achieved symptoms relief during the treatment and 13 patients achieved symptoms relief within 3 months after completion of RT. Table 2 displays the correlations between the patients’ RT responses and their clinical characteristics. The rate of CR after RT and BED2 (≥22 Gy vs. <22 Gy), underlying hematologic diseases (non-AML vs AML), sex, cytogenetics (normal vs abnormal), and BM status at the time of RT for MS (BM relapse vs BM remission) showed no significant correlations. Patients younger than 50 years of age had a marginally higher CR rate than those aged 50 years or older (83.3% vs. 37.6%, *P* = 0.06). Similarly, patients receiving BMT prior to RT had a marginally higher CR rate that those without previous BMT (100% vs. 50%, *P* = 0.05).

**Table 2:** Predictive factors associated with clinical CR to radiotherapy (n = 20)

**Characteristics**	**Complete response (No. of patients)**	**Partial response and stable disease (No. of patients)**	**Complete response rate**	**Crude odds ratio**	**95% Confidence interval**	P-value*
BED dose						
≥22 Gy	3	4	43%	4.44	0.62-32.07	0.14
<22 Gy	10	3	77%	1 (Referent)		
Underlying disease						
Non-AML	3	4	43%	4.44	0.62-32.07	0.17
AML	10	3	77%	1 (Referent)		
Age						
≥50 y	3	5	37.5%	8.33	1.03-67.14	0.06
<50 y	10	2	83.3%	1 (Referent)		
Gender						
Female	5	4	55.6%	2.13	0.33-13.81	0.64
Male	8	3	72.7%	1 (Referent)		
Cytogenetics						
Abnormal	6	4	60%	1.56	0.24-9.91	1
Normal	7	3	70%	1 (Referent)		
Previous BMT before RT						
Yes	6	0	100%	NA	NA	0.05
No	7	7	50%	1 (Referent)		
BM involvement at GS RT						
Yes	8	5	61.5%	1.56	0.22-11.37	1
No	5	2	71%	1 (Referent)		

*Abbreviations:* CR, complete remission; PR, partial response; SD, stable disease, BMT, bone marrow transplant; RT, radiotherapy; BM, bone marrow.

*Fisher’s exact test.

When using the GEE model to analyze all 43 irradiated lesions, we observed that BED2, age, sex, cytogenetics, and bone marrow status at the time of RT for MS showed no significant associations with CR (Table 3). Patients with AML had a trend toward higher CR rate than those with non-AML (OR = 5.436, 95% CI = 0.999-29.578, *P =* 0.05). According to the logistic regression model, BMT prior to RT was also associated with marginally significant increased rate of CR following RT (OR = 0.099, 95% CI = 0.0086-1.135, *P =* 0.06).

**Table 3:** Predictive factors associated with clinical complete remission to radiotherapy (43 lesions analyzed using the GEE model)

**Characteristics**	**Complete response (No. of patients)**	**Partial response and stable disease (No. of patients)**	**Complete response rate**	**Univariate odds ratio**	**95% Confidence interval**	***P-value***
BED dose						
≥22 Gy	6	4	60%	1.15	0.20-6.63	0.88
<22 Gy	21	12	63.64%	1 (Referent)		
Underlying disease						
Non-AML	3	6	33.33%	5.436	0.999-29.578	0.050
AML	24	10	70.59%	1 (Referent)		
Age						
≥50 y	9	5	64.29%	2.92	0.47-18.14	0.25
<50 y	18	11	62.07%	1 (Referent)		
Gender						
Female	7	5	58.33%	1.41	0.31-6.36	0.65
Male	20	11	64.52%	1 (Referent)		
Cytogenetics						
Abnormal	13	10	56.52%	1.35	0.27-6.83	0.72
Normal	14	6	70%	1 (Referent)		
Previous BMT before RT						
Yes	16	2	88.89%	0.10	0.01-1.14	0.06
No	11	14	44%	1 (Referent)		
BM involvement at GS RT						
Yes	16	8	66.67%	1.51	0.23-9.82	0.67
No	11	8	57.89%	1 (Referent)		

*Abbreviations:* CR, complete remission; PR, partial response; SD, stable disease; RT, radiotherapy; BMT, bone marrow transplant; BM, bone marrow.

### Clinicopathologic features and survival of MS patients without RT

Eighteen MS patients without RT served as control group. There were 10 (55.6%) men and 8 (44.4%) women with a median age of 17 years (range, 1–70 years) at diagnosis. The underlying hematologic diseases included AML in 14 (77.8%) patients, CML in 4 (22.2%) patients. The MS lesions included 5 (27.8%) skin, 4 (22.2%) bone, 2 (11.1%) orbital cavity, 1 (5.6%) brain, 1 (5.6%) breast, 1 (5.6%) cervix, 1 (5.6%) neck lymph node, 1 (5.6%) oral cavity, 1 (5.6%) ovary, and 1 (5.6%) uterus. Of the 18 patients, 9 (50%) had normal cytogenetics and 9 (50%) displayed abnormal chromosomes, among which t(9;22) (in 4 (22.2%) of 9 patients) and t(8;21)(q22;q22) (in 2 (11.1%) of 9 patients) were the common abnormality. Other observed abnormal cytogenetics included: t(9;11) in one patient, inv(7)(q22q36) in one patient, and del(7)(q32q36),del(18)(q21q23) in the other patient.

Treatments consisted of surgery alone in 1 (5.6%) patient, chemotherapy alone in 12 (66.4%) patients, a combination of surgery and chemotherapy in 2 (11.2%) patients, and bone marrow transplantation in 3 (16.8%) patients. CR was achieved in 16 (88.9%) of 18 patients and symptom relief was obtained in 17 (94.4%) of 18 patients. Of the 18 patients, 6 survived and 12 died. The median survival time was 8.5 months (range, 1–80 month) in all patients and 20.5 months (range, 6–80 month) in the surviving patients. The 1-year OS was 47.1%.

## Discussion

In this study, we evaluated clinicopathological features and treatment factors and their associations with RT responses in 20 patients with MS (in 43 RT courses), and also summarizes the clinicopathological features, cytogenetics, involved sites of MS, timing of RT, RT doses and fractions, and RT responses in published case reports (summarized in Table 4). Although our patients showed similar clinicopathologic features to those previously reported, we identified other diversified sites of MS involvement, including the breast [9-11], brain and spinal cord [12-15], cervix [16-19], paranasal sinus [20,21], and oral cavity [22,23]. We also observed previously unreported sites of involvement such as the nipple in men and the pituitary. Although the mechanism of MS formation is not fully understood, the leukemic cells homing to specific sites might be related to blast neural cell adhesion molecules (CD56) [2]. For example, high neural cell adhesion molecule expression in breast, testicular, ovarian, and gut tissue could explain these specific regions being common sites of MS involvement [24].

**Table 4:** Published case reports on radiotherapy for GS

**Study**	**Patient no.**	**Age**	**Cytogenetics**	**Underlying disease**	**Timing of GS emergence**	**Radiotherapy site**	**Dose fractionation**	**Response**
Lee *et al*. [25]	1	50	t(9;22)(q34;q11)	CML	Relapse after BMT, BM (-)	Clavicle and manubrium	15 Gy	CR
Taverna *et al.*[26]	1	62	-	AML-M4	Relapse after chemotherapy, BM also (+)	Left gluteal and the deeper pelvic muscles	20 Gy/10fx	PR
Au *et al.*[27]	1	24	del( 5)(q13; q33)	AML-M0	Relapse after BMT, BM (-)	Left breast	30 Gy	PD
Pulsoni *et al.*[28]	1	84	-	*De novo* sarcoma	At presentation, BM (-)	Skin	90 Gy/8fx	CR
Kasahara *et al.*[29]	1	47	der (1; 7)(q10; p 10)	Idiopathic myelofibrosis and *de novo* sarcoma	At presentation, BM (-)	Right submandibular tumor	30 Gy	CR
Buckland *et al.*[30]	1	35	Normal karyotype	*De novo* sarcoma	At presentation, BM (-)	C4-6 spine	32 Gy/16fx	CR
Lee *at al.*[31]	1	43	-	*De novo* sarcoma	At presentation, BM (-)	Maxillary gingiva	36 Gy	CR
Fleckenstein *et al.*[32]	1	73	Normal karyotype	AML	Found at initial presentation with BM (+)	Bilateral retrobulbar tumor	30 Gy/15fx	CR
Cozzi *et al.*[33]	1	39	Philadelphia positive	CML	Chronic phase	Left shoulder	21 Gy	PD
Nishimura *et al.*[34]	1	30	Add(3)(q27), t(8;21)(q22,q22)	AML-M2	Relapse after BMT, BM (-)	Right frontal intra/extracranial tumor	16 Gy	CR
Pelosini *et al.*[35]	1	25	t(8,21)with AML1-ETO expression.	AML-M0	Relapse after BMT, BM (-)	Left leg	40 Gy	CR
Rosenberg *et al.*[36]	1	8	del(7)(q22;q36),t(7;11)(p15;p15)	AML-M2	Relapse after BMT, BM also (+)	Bilateral synchronous epibulbar tumor	24 Gy/12fx	CR
Pitz *et al.*[37]	1	50	Normal karyotype	AML	Relapse after chemotherapy, BM also (+)	Uterus/ endometrium	30 Gy/10fx	CR
Kumar *et al.*[38]	1	10	-	*De novo* sarcoma	At presentation, BM (-)	Left orbit	24 Gy/12fx	CR
Vassiliou *et al.*[39]	1	40	-	*De novo* sarcoma	At presentation, BM (-)	Mediastinal lymph node	41.4 Gy/23fx	CR
Kozelj *et al.*[40]	1	52	t(8;21)	AML	At presentation, BM (+)	Heart	15 Gy/10fx	CR
Lee *et al.*[41]	1	25	45,X,-Y,del(2)(q21q31),t(5;11)(q31;q13),t(8;21)(q22;q22),t(10;19)(q22;q13.1)	*De novo* sarcoma	At presentation, BM (-)	Orbit	70 Gy	PR
Mauermann *et al.*[42]	1	70	Normal karyotype	*De novo* sarcoma	At presentation, BM (-)	Base of the skull to the upper thoracic region, including the brachial plexus	20 Gy/10fx	Good PR
Verra *et al*. [43]	1	45	inv(16)	AML-M4	Relapse after BMT, BM (-)	L4 to S3 spine	21 Gy/7fx	PR
Antic *et al.*[44]	1	24	Normal karyotype	*De novo* sarcoma	At presentation, BM (-)	Lumbosacral spine	40 Gy/20fx	CR
Mignano *et al.*[45]	1	20	-	AML-M2	Relapse after BMT, BM (-)	Left jaw	30 Gy/15fx	CR
						Heart	24 Gy/12fx	CR
Kim *et al.*[16]	1	30	-	*De novo* sarcoma	At presentation, BM (-)	Uterus/cervix	30 Gy	CR
Alvarez *et al.*[46]	1	41	CBFβ/MYH11 fusion and inv(16) (p13q22)	AML-M2	At initial presentation, BM also (+)	Small bowel, greater omentum and peritoneum	22.4 Gy/14fx	CR
Masetti *et al.*[47]	1	11	11q23 rearrangement (*MLL*-*AF10*)	AML-M5	In remission after allogeneic hematopoietic stem cell transplantation, BM (-)	L1 to S3 Epidural mass	20 Gy/16fx	Good PR at the end of RT
Chak *et al.*[5]	33 (54 courses of radiotherapy)	1.5 - 8 1	-	28 (84.8%) acute non-lymphocytic leukemia, 11 (15.2%) chronic leukiemia	-	33% bone, 31% soft tissue, 11% lymph node, 7% spinal cord, 6% brain, 11% other sites (Mediastinal mass, 2; pelvic mass, 1; pleural mass, 1 ; spleen, 1 ; porta hepatis, I)		<1000 rad, CR 18%, 1000–1999 rad, CR 43%; 2000–2900 rad, CR 86%; >3000 rad, CR 89%
Bakst *et al.*[9]	22 (33 courses of radiotherapy)	Median 34 (1–71)	-	AML 19 (86%), MDS 2 (9%), Isolated Chloroma 1 (5%)	32% in remission (43% of them have GS concomitantly with marrow relapse)	39% head and neck, 24% extremity, 9% spine, 9% brain, 6% genito-urinary, 6% breast, 3% pelvis, and 3% genitourinary	Median 20 (6–36) Gy/2 Gy (1.5–4 Gy)	CR 97%

*Abbreviations:* BM = bone marrow; CR = complete response; fx = fraction; Gy = gray; PD = progressive disease; PR = partial response.

In our cytogenetically abnormal patients, t(8;21)(q22;q22) was the most common cytogenetic change [48]. Limited previous reports exist on our identified cytogenetic abnormalities of t(2;14), t(9;22), and del(15)(q22q35) [11]. Systemic treatment outcome might be distinct among different cytogenetic groups. For example, in the study by Rollig et al., patients with t(8;21)(q22;q22) had good outcome following systemic treatment [49]. In our study, most patients had underlying AML disease. Of the non-AML patients, most had CML and a few had de novo sarcoma and MDS. In GEE analysis, we observed a trend that AML patients displayed higher CR rates than non-AML patients, which suggested that the underlying tumor behavior and biology of MS resulting from underlying diseases might differ. Further investigation to evaluate the radiobiological effects and elucidate the differential radiosensitivities of MS resulting from each underlying hematologic disease is warranted.

Another of our study findings was that BMT prior to RT resulted in marginally significant improvement of the CR rate in MS patients; however, the precise mechanism for the differential effects of RT on MS pre- and post-BMT are not well-understood. The incidence of patients developing MS after BMT is reportedly 0.2%-3.7% [50-52]. Myeloablative conditioning regimens of BMT often include high-dose busulfan/cyclophosphamide or cyclophosphamide in combination with total body irradiation. In allogeneic BMT, graft versus host interaction is an important factor in determining the success or toxicity of the transplant. Preclinical studies have demonstrated that radiation is involved in the recruitment of effector CD8+ T cells to nonlymphoid tissues and this effect enhances the graft-versus-leukemia (GVL) effect after allogeneic transplantation [53,54]. These findings may explain that the more favorable response of MS toward RT after bone marrow transplant might be resulted from RT-inducing GVL effect. The influences of marked immununological disturbance during transplantation and graft versus host/leukemia interaction on the effects of RT (which is highly dependent on immunological interactions among the tumor, normal cells, and tumor microenvironment) warrant further investigation.

Age is considered a prognostic factor in AML. In our study, patients younger than 50 years of age had a trend toward higher CR rates following RT than those aged 50 years or older (83.3% vs 37.6%, *P* = 0.06). Kantarjian et al. reported reduced anthracycline sensitivity and Ras, Src, and tumor necrosis factor pathway activation in older patients with AML [55]. These deregulated signaling pathway variations may explain the poor response to RT in older patients with MS.

Although RT generally yields high CR rate, the optimal dose for MS has yet to be determined. Chak et al. [5] investigated the relationship between RT dose and response in 33 MS patients (54 RT courses), observing that the CR rate was closely associated with RT dose (CR rate: <10 Gy, 18%; 10–19.99 Gy, 43%; 20–29.99 Gy, 86%; >30 Gy, 89%). In their study on 22 patients with MS (33 RT courses) who received a median dose of 20 Gy (range 6–36 Gy) in fractions of 1.5-4 Gy, Bakst et al. showed that RT resulted in excellent disease control and minimal morbidity [9]. The authors recommended a low-dose regimen of 24 Gy in 12 fractions to irradiate MS. Our findings are consistent with the two mentioned large series reports, and demonstrate excellent local control in our 20 MS patients (43 RT courses), who received a median dose of 20 Gy (range 6–35 Gy) in fractions of 1.5-3.5 Gy. Although our results indicated that BED2 had nonsignificant effects on CR rate (≥22 Gy vs <22 Gy), most previous cases series have reported good local control of MS in low to moderate RT doses between 20 Gy and 30 Gy (Table 4). The main mechanism of radiation damage to leukemic cells is apoptosis. For high-dose irradiation, the D_0_ of p53-deficient HL-60 leukemic cells is 2.2 Gy, and the D_0_ of p53 wild MOLT-4 leukemic cells is 0.87 Gy [56]. According to radiobiological and clinical data, a 20–30 Gy radiation dose might be sufficient to achieve good local control for MS [57].

## Conclusion

In summary, in this study, we evaluated the clinicopathological features and radiotherapeutic responses of 20 MS patients with 43 lesions, and identified unusual sites of MS presentation, including the nipple in men and the pituitary. Diversified cytogenetic abnormalities can occur in MS; however, the most common MS-associated cytogenetic change is t(8;21)(q22;q22). We further identified that the CR rate is optimal using moderate RT doses between 20 Gy and 30 Gy with conventional fractionation. Younger age, BMT prior to RT, and AML patients had marginally significant trend toward higher CR rate in MS.

### Consent

Informed consent is waived.

## Competing interests

The authors declare that they have no competing interest.

## Authors’ contribution

WYC, SHK and ALC contributed to the study design; WYC, WCW, KHL, JLT, THF, ALC and SHK treated patients; WYC and SHk collected clinical data; WYC, CHC, HHL and SHK were involved in data analysis and interpretation; WYC, WCW, KHL and SHK wrorte the manuscript; and all authors revised and approved the final manuscript.
